# Psychotropic Effects of an Alcoholic Extract from the Leaves of* Albizia zygia* (Leguminosae-Mimosoideae)

**DOI:** 10.1155/2017/9297808

**Published:** 2017-10-03

**Authors:** Patrick Amoateng, Dorcas Osei-Safo, Kennedy Kwami Edem Kukuia, Samuel Adjei, Obed Awintuma Akure, Constance Agbemelo-Tsomafo, Shirley Nyarko Adu-Poku, Kenneth Yaw Agyeman-Badu

**Affiliations:** ^1^Department of Pharmacology & Toxicology, School of Pharmacy, College of Health Sciences, University of Ghana, P.O. Box LG 43, Legon, Accra, Ghana; ^2^Department of Chemistry, School of Physical and Mathematical Sciences, College of Basic and Applied Sciences, University of Ghana, P.O. Box LG 56, Legon, Accra, Ghana; ^3^Department of Animal Experimentation, Noguchi Memorial Institute for Medical Research (NMIMR), College of Health Sciences, University of Ghana, P.O. Box LG 581, Legon, Accra, Ghana

## Abstract

**Background:**

* Albizia zygia* is used in Ghanaian traditional medicine for the management of mental disorders. The present study tested the hypothesis that an extract of the leaves of* Albizia zygia* (AZE) may possess antipsychotic and antidepressant properties.

**Method:**

The novelty- and apomorphine-induced locomotor and rearing behaviours of AZE in mice were explored in an open-field observational test system. The effects of AZE in apomorphine-induced cage climbing test, extract-induced catalepsy, and haloperidol-induced catalepsy on mice were also investigated. Lastly, the forced swimming and tail suspension tests in mice were employed to screen the possible antidepressant effects of AZE.

**Results:**

AZE (100–3000 mg/kg) showed signs of central nervous system (CNS) depression under observation, with no lethality, 24 h after treatment in mice. AZE (100–1000 mg/kg) produced a significant decrease in the frequency of novelty- and apomorphine-induced locomotor activities in mice. The extract also significantly decreased the frequency and duration of apomorphine-induced climbing activities in mice. AZE, while failing to produce any cataleptic event in naïve mice, significantly enhanced haloperidol-induced catalepsy at a dose of 1000 mg/kg. However, AZE did not produce any significant antidepressant effects in the test models employed.

**Conclusion:**

The extract of* Albizia zygia* exhibited an antipsychotic-like activity in mice.

## 1. Introduction

*Albizia zygia *(family Leguminosae-Mimosoideae) is a medium-sized semideciduous tree that can grow up to about 30 m high and widespread in tropical Africa, known locally in Ghana as* “Okuro”* among the Akans [1]. The wood obtained from the tree is used for indoor construction, veneer and plywood, boat and ship building, implements (e.g., pestles, hoe-handles), matches, and vehicle bodies. In the Ghanaian traditional medicine, the leaves of* Albizia zygia* are used in the management of mental troubles [2]. In other indigenous African traditional health systems, the bark sap is instilled in the eyes to treat eye infections and the bark decoction is also useful for the treatment of bronchial diseases, malaria, fever, and female sterility [3]. The pounded bark is applied topically to treat yaws, sores, wounds, and toothache. Leaf decoctions are administered to treat fever and diarrhoea. Ground roots of the plant are added to food to treat cough and as an expectorant. The root bark juice is also used on wounds to promote healing [3]. In Nigeria, some communities use the plant for the treatment of waist pain [4] and in Cameroon, decoction of the leaves and stem is used in the treatment of boils, diarrhoea, male sexual impotence, oedema, and fracture [5, 6].

Three flavonoids (4′,7-dihydroxyflavanone, 3′,4′,7-trihydroxyflavone, and 3-*O*-methylfisetin (3′,4′,7-trihydroxy-3-methoxyflavone)) isolated from* Albizia zygia* were tested against* Plasmodium falciparum* K1 and only 3′,4′,7-trihydroxyflavone showed potent antimalarial activity (IC_50_ 0.078 *μ*g/ml) [7]. Hexane and methanol extract of* Albizia zygia* has demonstrated* in vitro* free radical scavenging and antioxidant effects [8]. The aqueous methanolic stem bark extract of* Albizia zygia* has been found to possess analgesic activity and low subacute toxicity [9]. Recently, the antipyretic and analgesic properties of the plant extracts have been demonstrated [10, 11].

In African countries such as Ghana, up to 80% of the population use traditional medicine for their primary health care, and these treatment options are less expensive and easily accessible [12]. The drug discovery paradigm involving the investigation of medicinal plants has produced successful results such as the isolation of reserpine from* Rauwolfia serpentina* which is known to have antipsychotic properties [13–15], in addition to its better known antihypertensive effect.

In our quest to provide pharmacological validation of the traditional use in mental health, the antipsychotic and antidepressant effects of an ethanolic extract of the leaves of* Albizia zygia* in mice were evaluated.

## 2. Materials and Methods

### 2.1. Chemicals and Reagents

Chlorpromazine hydrochloride (Renandin, France) and haloperidol (STEROP, Belgium) were obtained from local suppliers, whereas apomorphine hydrochloride was obtained from Macfarlan Smith Ltd., Scotland, UK.

### 2.2. Plant Collection and Extraction

Fresh leaves of the plant were collected from the Aburi Botanical Gardens, Aburi, Ghana, in March 2015 by a curator at the Ghana Herbarium, Department of Plant and Environmental Biology, University of Ghana, Legon, Accra, who identified and authenticated them. A voucher specimen (PA03/UGSOP/GH15) was deposited at the herbarium. The leaves were air-dried for seven days and powdered. A weighed amount of the powder was cold-macerated with 70% v/v of ethanol in water. The ethanolic extract was then evaporated to dryness under reduced pressure, labelled as AZE, and stored in a desiccator.

### 2.3. Qualitative Phytochemical Screening of AZE

AZE was screened for the presence of alkaloids, saponins, tannins, flavonoids, and other phytochemicals using standard qualitative colorimetric methods [16].

### 2.4. Animals and Husbandry

Female Imprint Control Region (ICR) mice (weighing 20–30 g), 6–8 weeks old, were obtained from the Department of Animal Experimentation, Noguchi Memorial Institute for Medical Research (NMIMR), University of Ghana, Legon, Accra. The mice were accommodated in groups of five in stainless steel cages (dimensions: 34 cm × 47 cm × 18 cm) with soft wood chips as bedding and kept under laboratory conditions (temperature 22 ± 2°C, relative humidity 60–70%, and 12-hour light-dark cycle). The mice were fed with normal commercial pellet diet (AGRIMAT, Kumasi) and given water ad libitum. All the behavioural testing was conducted from 8:00 to 15:00 GMT.

### 2.5. Irwin's Test

Irwin's test for primary observation of AZE in mice was done as previously described [17]. Briefly, mice were treated with AZE (30, 100, 300, 1000, and 3000 mg/kg, p.o.) and vehicle (distilled water, 10 ml/kg, p.o.) as control. The mice were then observed 15, 30, 60, 120, and 180 min after administration of the extract/distilled water. The observation was repeated 24 hours later. The parameters, death, convulsions, sedation, excitation, jumping, abnormal gait (rolling, tiptoe), motor incoordination, loss of grasping, akinesia, catalepsy, loss of traction, loss of balance, fore-paw treading, writhing, piloerection, stereotypies (sniffing, chewing, and head movements), head-twitches, scratching, altered respiration, aggression, altered fear, altered reactivity to touch, ptosis, exophthalmia, loss of righting reflex, loss of corneal reflex, analgesia, defecation/diarrhoea, salivation, lacrimation, and pupil diameter (myosis/mydriasis), were recorded when exhibited.

### 2.6. Open-Field Test: Novelty-Induced Behaviours

The extract/drug-induced locomotor and rearing behaviours were determined in an open-field observation box (dimensions: 25 cm × 25 cm × 30 cm) made of transparent Perspex. The base of the maze has 16 squares (6.5 cm × 6.5 cm) demarcated with a nontoxic permanent marker. The arena of the open field was also designated as (i) corner (one of the four corner squares); (ii) periphery (the squares along the walls); or (iii) centre (the 4 inner squares). In the investigation, groups of mice were treated with AZE (100, 300, and 1000 mg/kg, p.o.), chlorpromazine 1 mg/kg (i.p.), or vehicle (distilled water, 10 ml/kg, p.o.). The selected doses of the extract were based on the findings from Irwin's test described above. Thirty minutes later, the mice were placed individually into the open-field observational box and their behaviour was recorded for 5 min using a camcorder (Everio™ Model, GZ-MG 130 U, JVC, Tokyo, Japan) suspended above the maze with the aid of a stand. Rearing behaviour was recorded when a mouse stood on its hind limbs and placed the forelimbs against the wall of the observation cage (supported rearing) or in free air (unsupported rearing). The number of rears (both supported and unsupported) was tracked for 5 min. In addition, the number of times a mouse crosses any of the lines demarcating the base of the maze (i.e., line-crossing) was counted as a representation of locomotor activity. The total frequency and duration spent in the corner and periphery or central portions of the observation set-up by the mice were also recorded.

### 2.7. Open-Field Test: Apomorphine-Induced Behaviours

In the open-field paradigm, as described above, mice were pretreated with AZE (100, 300, or 1000 mg/kg, p.o.), chlorpromazine (0.1, 0.3, or 1.0 mg/kg, i.p.), or vehicle (distilled water, 10 ml/kg, p.o.) and 30 min later, they received apomorphine (2 mg/kg, i.p.). The mice were then placed in the open-field test chamber. A no-apomorphine vehicle group was also included. The events were recorded with a camcorder for 30 min and frequency of rearing and line-crossing behaviours was tracked for each mouse. Also the total frequency and duration the mice spent in the corner, periphery, or central portions of the observation set-up were recorded.

### 2.8. Cage Climbing Test

The method as described previously was adopted [18]. In brief, mice were treated with AZE (100, 300, and 1000 mg/kg, p.o.), haloperidol (0.1, 0.3, and 1 mg/kg, i.p.), or vehicle (distilled water, 10 ml/kg, p.o.). After 30 min, they were injected with apomorphine (2 mg/kg, i.p.). The mice were then immediately placed individually into an all wire-meshed cage (mesh size: 1 cm × 1 cm; dimensions = 27 cm × 20 cm × 20 cm) and their climbing behaviour recorder with a camcorder which was placed above the cage for 30 min after apomorphine injection. The frequency and duration of climbing were tracked from the recorded video.

### 2.9. Catalepsy: Extract/Drug-Induced Motor Effect

An extract/drug-induced cataleptic test in mice was conducted as previously described [19, 20]. The set-up used in this test consists of a Perspex rod elevated with support to a height of 3.5 cm. Mice pretreated with the AZE (100, 300, and 1000 mg/kg, p.o), haloperidol (0.1, 0.3, and 1.0 mg/kg, i.p.), or vehicle were tested individually on this set-up 15, 30, and 60 min after treatment. The time a mouse spent when placed on the rod with its forepaws was recorded. The test ended when the animal removed its forepaws from the rod unto the floor or climbed the rod.

### 2.10. Potentiation/Inhibition of Haloperidol-Induced Catalepsy

The effect of AZE on haloperidol-induced catalepsy was performed as previously described [20, 21]. Mice were pretreated with AZE (100, 300, or 1000 mg/kg, p.o.) or vehicle (distilled water, 0.01 ml/kg, p.o.). Thirty minutes later, each mouse was treated with haloperidol 1 mg/kg (i.p.) and tested for catalepsy as described above.

### 2.11. Forced Swimming Test (FST)

This procedure was carried out using published methods with modifications [22–24]. Briefly, mice (*n* = 5–10) were pretreated with AZE (100, 300, and 1000 mg/kg, p.o.), fluoxetine (3, 10, and 30 mg/kg, p.o.), or vehicle (10 ml/kg of normal saline, p.o.), one hour (p.o.) or 30 min (i.p.) before being placed individually in polypropylene cylinders (height 25 cm, diameter 10 cm) containing water to a height of 15 cm, maintained at 32°C. With a public domain software JWatcher, version 1.0 (University of California, Los Angeles, USA, and Macquarie University, Sydney, Australia), behavioural assessment was measured during the 5-minute test period. Immobility behaviours (floating passively in the water without active movement except for twitches, shivers, or corrective wall-bouncing), swimming behaviours (movement of the hind limbs or tail resulting in a propulsive force or swimming motions, more than necessary, to solely maintain their head above water), and climbing behaviours (active movements in and out of the water with forepaws, usually directed against the walls) of the mice were scored.

### 2.12. Tail Suspension Test

The tail suspension test (TST) was carried out according to previous descriptions with modifications [24, 25]. Animals were randomly grouped in a similar fashion as in the FST and given AZE (100, 300, and 1000 mg/kg, p.o.), fluoxetine (3, 10, and 30 mg/kg, p.o.), or vehicle (10 ml/kg of normal saline, p.o.), one hour after oral administration and 30 min after intraperitoneal injection. Mice were individually suspended by the tail by tape wrapped around the distal third of the tail such that the body hung on a horizontal ring stand bar is raised 30 cm above the floor. Test sessions lasted 5 min and were videotaped. Behaviours rated were as follows: (1) immobility: a mouse was judged to be immobile when it performs no active behaviour, (2) swinging: a mouse was judged to be swinging when it continuously moved its paws in the vertical position while keeping its body straight and/or it moved its body from side to side, and (3) curling: a mouse was judged to be curling when it engaged in active twisting movements of the entire body.

## 3. Results

### 3.1. Qualitative Phytochemical Screening of AZE

Alkaloids, flavonoids, tannins, saponins, and anthraquinone glycosides were found to be present in AZE.

### 3.2. Irwin's Test

AZE (100, 300, 1000, and 3000 mg/kg) showed signs of CNS depression under observation. Extract-treated mice (at all dose levels) showed dose and time dependent sedation which lasted 60 minutes. There were no signs of convulsion and no mouse died after 24 hours suggesting LD_50_ is above 3000 mg/kg.

### 3.3. Open-Field Test: Novelty-Induced Behaviours

**Figure 1:**
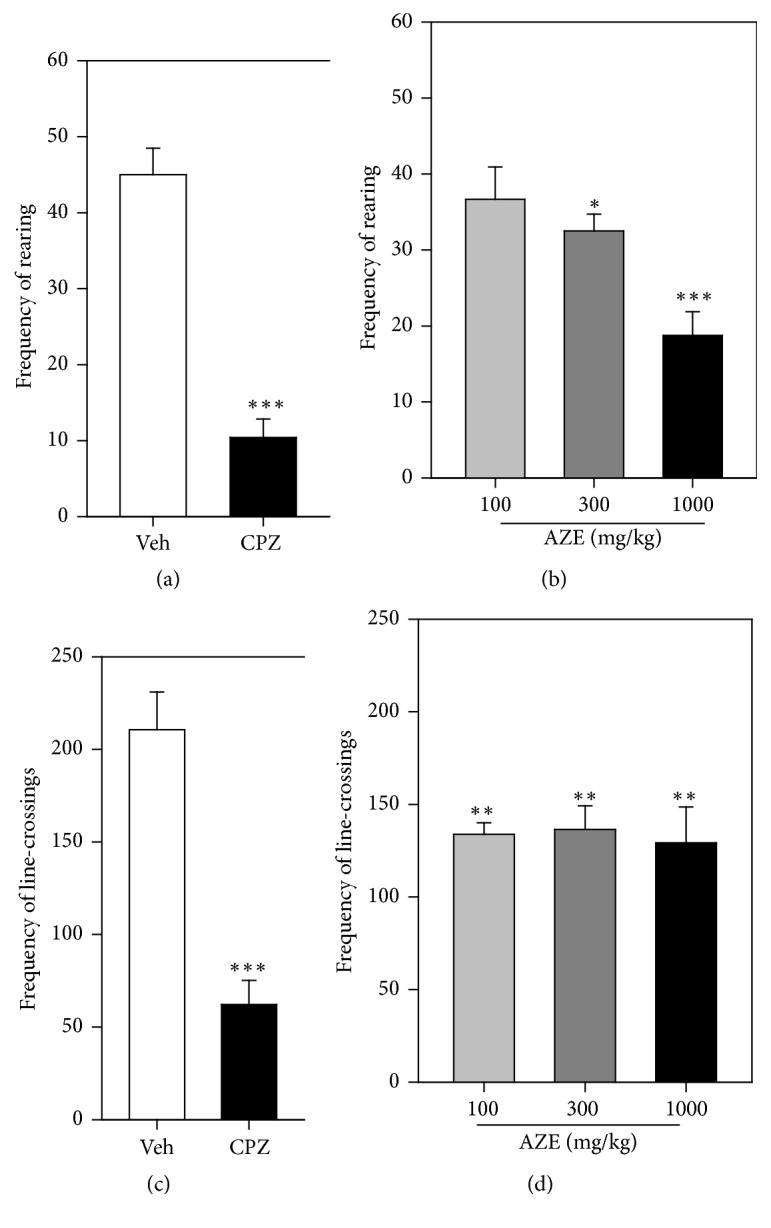
Frequency of rearing ((a) and (b)) and line-crossings ((c) and (d)) of mice treated with chlorpromazine (CPZ; 1 mg/kg, i.p.), AZE (100, 300, and 1000 mg/kg, p.o.), and vehicle (Veh) when observed for 5 min in an open-field paradigm. Data are mean ± SEM (*n* = 5). ^*∗*^*P* ≤ 0.05, ^*∗∗*^*P* ≤ 0.01, and ^*∗∗∗*^*P* ≤ 0.001 compared with vehicle group (one-way ANOVA followed by a Dunnett's multiple comparison post hoc test).

**Figure 2:**
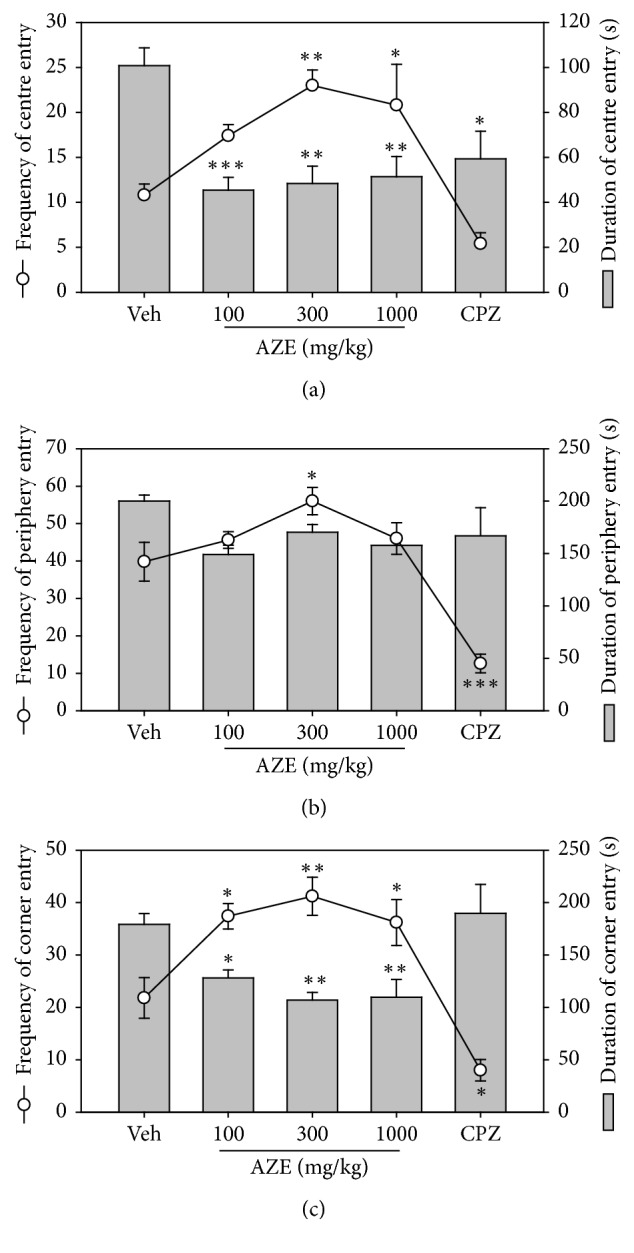
Effects of AZE (100–1000 mg/kg) on the frequency (line graph) and duration (column graphs) of centre entry (a), periphery entry (b), and corner entry (c). Each point represents mean ± SEM (*n* = 5). ^*∗*^*P* < 0.05, ^*∗∗*^*P* < 0.01, and ^*∗∗∗*^*P* < 0.001, compared to vehicle-treated group (one-way ANOVA followed by Dunnett's post hoc test).

AZE (100, 300, and 1000 mg/kg, p.o.) significantly decreased the frequencies of rearing (*P* < 0.0001,* F*_4,15_ = 22.82, Figure 1) and line-crossing (*P* < 0.0001,* F*_3,16_ = 11.83, Figure 1) in the mice treated, in comparison with the vehicle-treated group. The decrease in the frequency of the rearing behaviour was dose-dependent. In the 5 min test period, the AZE (100, 300, and 1000 mg/kg, p.o.) significantly increased the frequency of centre, periphery, and corner entry (*P* = 0.0002,* F*_4,20_ = 9.366; Figure 2(a), *P* < 0.0001,* F*_4,20_ = 19.33; Figure 2(b), and *P* < 0.0001,* F*_4,20_ = 16.62; Figure 2(c), resp.) compared to the vehicle-treated group. The extract significantly decreased the time spent by mice in the centre and corner (*P* = 0.0013,* F*_4,17_ = 7.268; Figure 2(a) and *P* = 0.0008,* F*_4,20_ = 7.861; Figure 2(c), resp.) of the open field. Also, AZE produced a decrease in the time spent by mice in the periphery (*P* = 0.1469,* F*_4,20_ = 1.917; Figure 2(b)) of the open field, but this was not significant.

### 3.4. Open-Field Test: Apomorphine-Induced Behaviours

**Figure 3:**
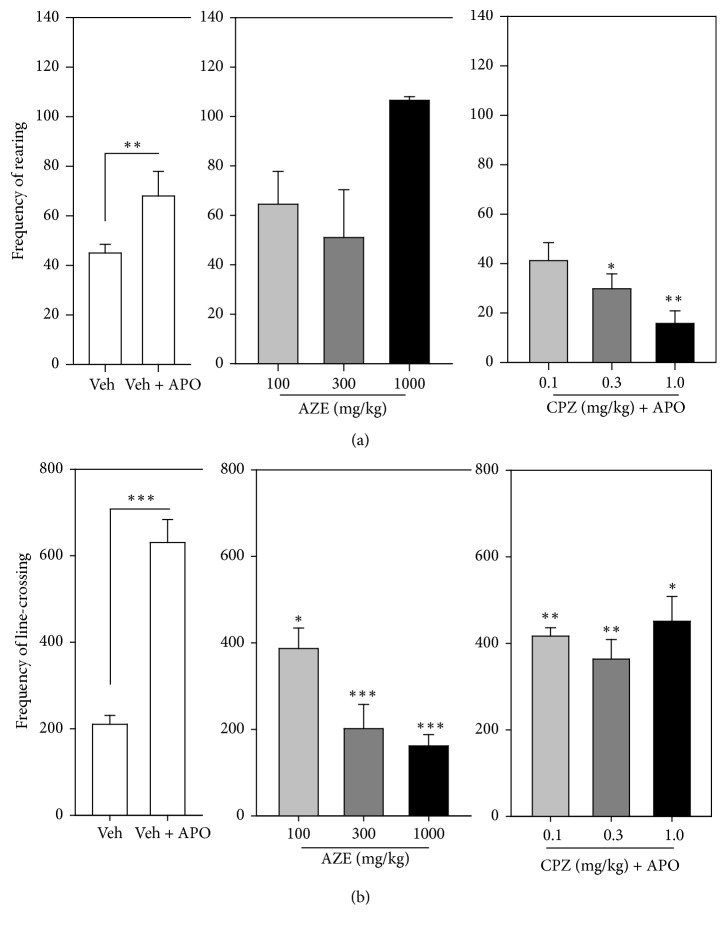
Effect of AZE (100, 300, and 1000 mg/kg, p.o.), CPZ (0.1, 0.3, and 1.0 mg/kg, i.p.), and vehicle (Veh) (distilled water, 0.01 ml/kg, p.o.) on the frequency of rearing (a) and line-crossings (b) of mice, 30 min after apomorphine (APO) (2 mg/kg, i.p.) treatment in an open-field paradigm. Data are mean ± SEM (*n* = 5). ^*∗*^*P* ≤ 0.05, ^*∗∗*^*P* ≤ 0.01, and ^*∗∗∗*^*P* ≤ 0.001 compared with Veh + APO group (one-way ANOVA followed by a Dunnett's multiple comparison post hoc test).

**Figure 4:**
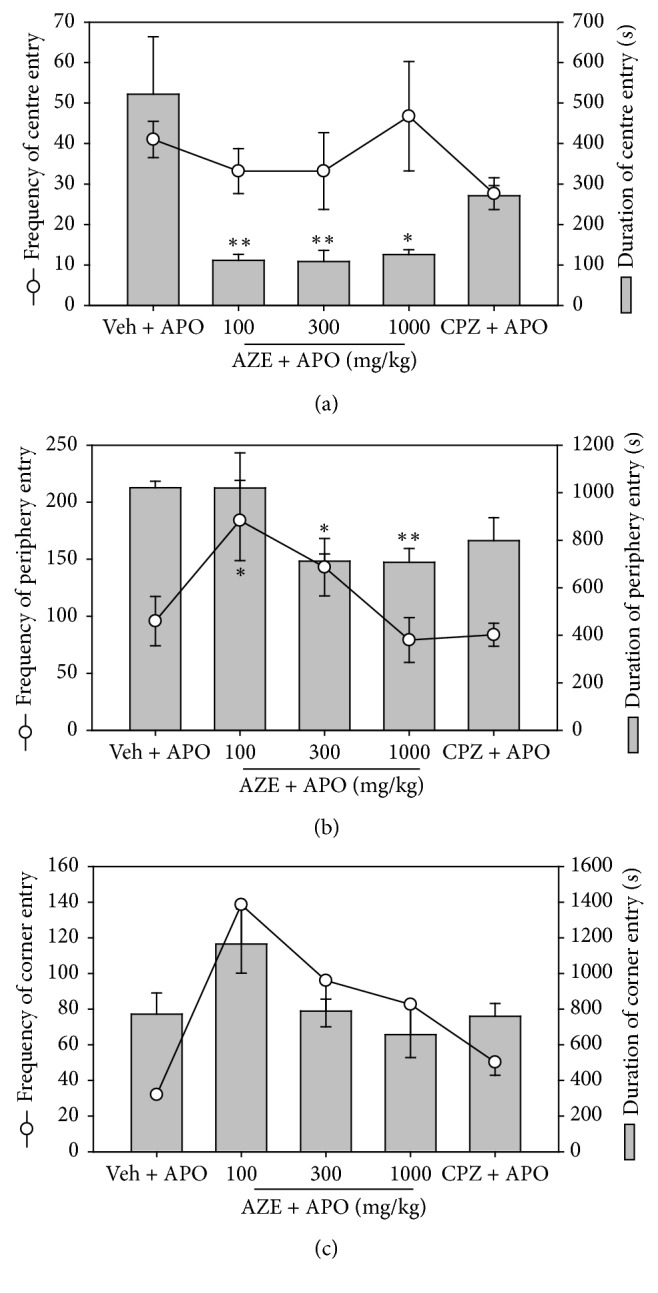
Effect of AZE (100, 300, and 1000 mg/kg, p.o.), CPZ (1.0 mg/kg, i.p.), and vehicle (Veh) (distilled water, 0.01 ml/kg, p.o.) on the frequency (line graph) and duration (column graphs) of centre entry (a), periphery entry (b), and corner entry (c), 30 min after apomorphine (APO) (2 mg/kg, i.p.) treatment in an open-field paradigm. Data are mean ± SEM (*n* = 5). ^*∗*^*P* ≤ 0.05 and ^*∗∗*^*P* ≤ 0.01 compared with Veh + APO group (one-way ANOVA followed by a Dunnett's multiple comparison post hoc test).

Apomorphine (2 mg/kg, i.p.) produced a characteristic increase in rearing and locomotor activity in the vehicle-treated group when compared with the vehicle-alone-treated group of mice (i.e., no apomorphine) (Figures 3(a) and 3(b)). There was a significant and dose-dependent decrease in the frequency of line-crossing behaviour (*P* = 0.0002,* F*_3,9_ = 20.90, Figure 3(b)) in the mice treated with AZE in comparison to the vehicle-treated mice. However, AZE failed to elicit any significant change in the frequency of rearing in the mice treated with it (*P* = 0.2217,* F*_3,10_ = 1.741, Figure 3(a)). Chlorpromazine significantly decreased apomorphine-induced rearing (*P* = 0.0065,* F*_3,15_ = 6.067, Figure 3(a)) and locomotor activity (*P* = 0.007,* F*_3,12_ = 6.58, Figure 3(b)) in mice that were pretreated with it. The decrease in rearing activity by CPZ was dose-dependent, whereas that of the line-crossing was not. In comparison to the vehicle-treated group, AZE (100, 300, and 1000 mg/kg, p.o.) decreased, but not significantly, the frequency of centre entry (*P* = 0.5044,* F*_4,18_ = 0.8636; Figure 4(a)) but significantly reduced the time spent by mice in the centre (*P* = 0.0077,* F*_4,15_ = 5.228; Figure 4(a)) of the open field. The extract significantly increased the frequency of periphery entry (*P* = 0.0228,* F*_4,17_ = 3.761; Figure 4(b)) and significantly decreased the time spent by animals in the periphery (*P* = 0.0054,* F*_4,17_ = 5.400; Figure 4(b)) of the open field. AZE also increased, but not significantly, the frequency of entries into the corner (*P* = 0.1168,* F*_4,18_ = 2.146; Figure 4(c)) but produced no significant change in the time spent by mice in the corner (*P* = 0.1290,* F*_4,19_ = 2.043; Figure 4(c)) of the open field.

### 3.5. Cage Climbing Test

**Figure 5:**
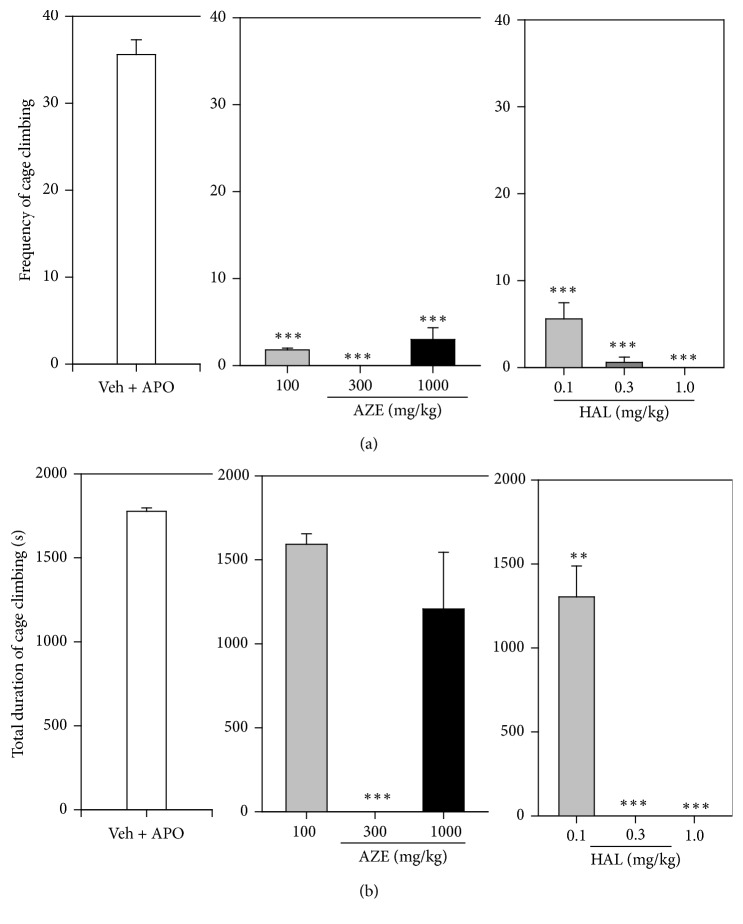
Effect of AZE (100, 300, and 1000 mg/kg, p.o.), HAL (0.1, 0.3, 1.0 mg/kg, i.p.), and vehicle (Veh) on the total frequency and duration of cage climbing of mice, 30 min after apomorphine (APO) (2 mg/kg, i.p.) treatment. Data are mean ± SEM (*n* = 5). ^*∗∗*^*P* ≤ 0.01 and ^*∗∗∗*^*P* ≤ 0.001 compared with Veh + APO group (one-way ANOVA followed by a Dunnett's multiple comparison post hoc test).

Apomorphine induced a characteristic increase in the total frequency (Figure 5(a)) and duration (Figure 5(b)) of climbing of the wire-meshed cage by the animals. AZE (100, 300, and 1000 mg/kg, p.o.) significantly reduced the total frequency (*P* < 0.0001,* F*_3,16_ = 247.3; Figure 5(a)) and duration (*P* < 0.0001,* F*_3,12_ = 32.79; Figure 5(b)) of cage climbing in the treated mice, but not in the dose-dependent manner. Haloperidol (HAL) (0.1, 0.3, and 1 mg/kg, i.p.) significantly and dose-dependently reduced the total frequency (*P* < 0.0001,* F*_3,16_ = 172.1, Figure 5(a)) and duration of cage climbing (*P* < 0.0001,* F*_3,15_ = 146.7, Figure 5(b)). Comparison of the ED_50_ values of AZE and haloperidol revealed that HAL was more potent than AZE (Table 1). However, the effect of AZE at dose of 300 mg/kg, p.o., which was the most potent (*P* < 0.0001) extract dose in reducing climbing behaviour, was comparable to haloperidol at dose 1 mg/kg, i.p.

### 3.6. Catalepsy: Extract/Drug-Induced Motor Effects

AZE (100, 300, and 1000 mg/kg) did not produce any cataleptic behaviour (*P* = 0.23,* F*_3,16_ = 1.753, Table 2) in the mice treated. Haloperidol (0.1, 0.3, and 1 mg/kg), however, exhibited a significant and dose-dependent cataleptic behaviour in the treated mice (*P* = 0.01,* F*_3,16_ = 5.209, Table 2).

### 3.7. Potentiation/Inhibition of Haloperidol-Induced Catalepsy

**Figure 6:**
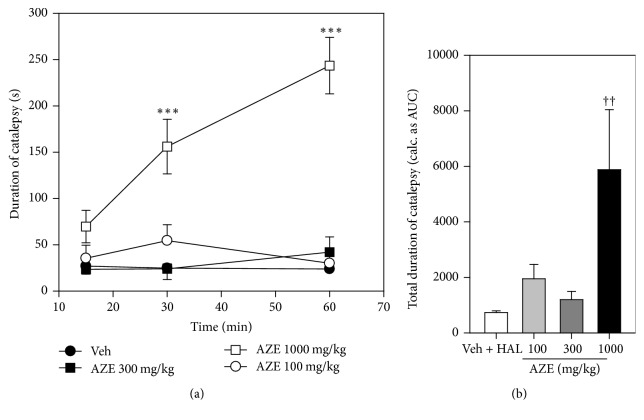
Effects of AZE on haloperidol- (HAL-) induced catalepsy in mice. Paneled graph (a) is the time-course effects 15, 30, and 60 min after haloperidol administration. Column graph (b) is the total duration of catalepsy (calculated as AUCs from the time-course graphs). Data are mean ± SEM (*n* = 5). ^*∗∗∗*^*P* ≤ 0.001 compared with vehicle group (two-way ANOVA followed by Bonferroni post hoc test). ^††^*P* ≤ 0.01 compared with vehicle group (one-way ANOVA followed by a Dunnett's multiple comparison post hoc test).

AZE 100 and 300 mg/kg inhibited the cataleptic effect of haloperidol (1 mg/kg) throughout the 60 min after haloperidol treatment. However, AZE 1000 mg/kg increased significantly the haloperidol-induced catalepsy, 30 and 60 min after haloperidol treatment (Figure 6(a)). Overall, there was a significant difference (*P* = 0.0087,* F*_3,12_ = 6.200, Figure 6(b)) between the AZE- and vehicle-treated mice in this test and only AZE 1000 mg/kg significantly increased the haloperidol-induced cataleptic behaviour (*P* < 0.01, Figure 6(b)).

### 3.8. Forced Swimming Test

**Figure 7:**
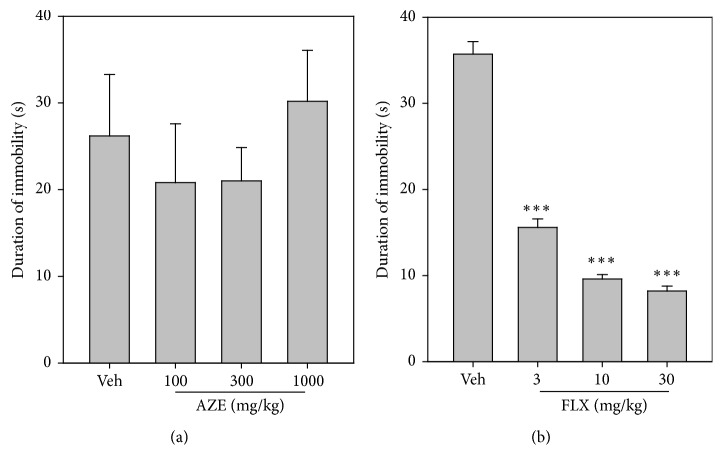
Effects of AZE (100–1000 mg/kg) (a) and FLX (3–30 mg/kg) (b) on the duration of immobility in the FST. Each point represents mean ± SEM (*n* = 5). ^*∗∗∗*^*P* < 0.001, compared to vehicle-treated group (one-way ANOVA followed by Dunnett's post hoc test).

AZE (100, 300, and 1000 mg/kg, p.o.) administered 60 min before the test period produced no significant reduction in the duration of immobility (*P* = 0.6480,* F*_3,16_ = 0.5618; Figure 7(a)) of mice in the FST. Fluoxetine (FLX) (3, 10, and 30 mg/kg, p.o.) significantly reduced the duration of immobility (*P* < 0.0001,* F*_3,18_ = 151.1; Figure 7(b)) of mice in a dose-dependent manner relative to the vehicle-treated group.

### 3.9. Tail Suspension Test

**Figure 8:**
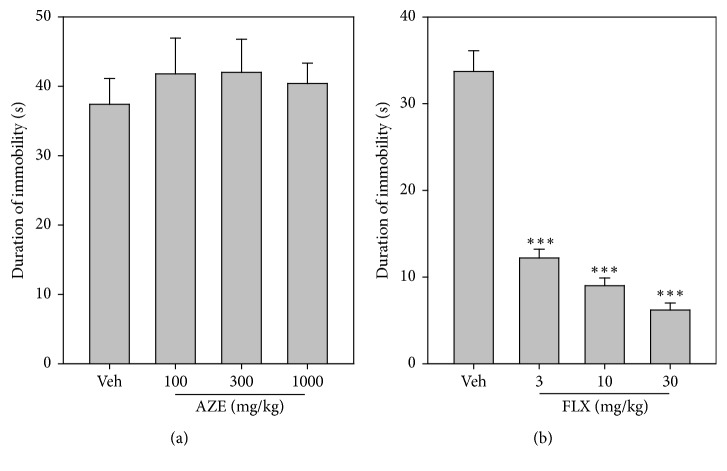
Effects of AZE (100–1000 mg/kg) (a) and FLX (3–30 mg/kg) (b) on the duration of immobility in the TST. Each point represents mean ± SEM (*n* = 5). ^*∗∗∗*^*P* < 0.001, compared to vehicle-treated group (one-way ANOVA followed by Dunnett's post hoc test).

AZE (100, 300, and 1000 mg/kg, p.o.) administered 60 min prior to the test period failed to produce any reduction in the duration of immobility (*P* = 0.8600,* F*_3,16_ = 0.2502; Figure 8(a)) of mice in the TST. Fluoxetine (FLX) (3, 10, and 30 mg/kg, p.o.), as expected, significantly and dose-dependently reduced the duration of immobility (*P* < 0.0001,* F*_3,18_ = 61.74; Figure 8(b)) of mice with respect to the vehicle-treated group.

## 4. Discussion

The present study describes an antipsychotic and antidepressant potential of a hydroethanolic extract of the leaves of* Albizia zygia* (AZE) in murine models of psychosis and depression. The experimental procedures were done to rationalise the traditional uses of the plant in mental health. The behavioural effects of* Albizia zygia* in mice were examined. Mice were used as the experimental subject because they are similar to humans at the anatomical, cellular, biochemical, and molecular level and also share with humans similar brain functions such as anxiety, hunger, circadian rhythm, aggression, memory, sexual behaviour, and other emotional responses [26]. The findings of this study provide initial evidence that AZE possesses antipsychotic-like effects. However, AZE seem to be devoid of antidepressant-like activity, as seen from the findings of the effects of AZE on the murine models of depression employed.

A preliminary investigation of the effects of the AZE in Irwin's tests, novelty-induced rearing, and locomotor activity demonstrates that the extract is sedative and that it decreases exploratory behaviour of the pretreated mice. These effects exhibited by AZE do not necessarily suggest an antipsychotic activity, though most antipsychotic agents produce similar effects in animals [27].

Stimulation of cerebral dopaminergic activity can produce psychosis de novo in some patients and psychosis has long been associated with increased cerebral dopaminergic activity. This role of dopamine in psychosis is supported by biochemical findings, clinical and imaging studies [28–31]. The clinical efficacy of most antipsychotic drugs, especially those effective against the positive psychotic symptoms such as hallucinations and delusions, may be linked to dopamine D_2_ receptor antagonism [32]. Murine models of psychosis used for screening antipsychotic drugs are based on the neurochemical hypothesis of schizophrenia, involving mainly the neurotransmitters, dopamine and glutamate [33]. The dopamine-based models usually use apomorphine, a direct dopamine agonist, or amphetamine, a drug that increases the release of dopamine and blocks its reuptake [34]. In this study, psychosis in the murine models was induced by administration of apomorphine which, being a nonselective dopamine agonist, elicits behavioural responses in animals by activating dopamine D_1_ and D_2_ receptors in the brain [35, 36]. Activation of these dopamine receptors is known to increase locomotor activity [37], stereotyped behaviours [38], rearing/grooming [39], and cage climbing behaviours [40, 41].

The apomorphine-induced cage climbing test is a widely used test for screening antipsychotic drugs [18, 40, 42]. It has predictive validity for antipsychotic drugs that normalize the hyperactivity and stereotypic behaviour in that almost all antipsychotics dose-dependently antagonize apomorphine-induced cage climbing behaviour of mice [42]. Mice treated with low doses of apomorphine tend to adopt a vertical position and try to climb walls. Apomorphine induces climbing behaviour in mice by activating both dopamine D_1_ and D_2_ receptors in the striatum [42]. Activation of either dopamine D_1_ or D_2_ receptors does not induce climbing behaviour; instead, activation of both receptors is required to produce climbing behaviour [43]. Therefore, it follows that antagonism of apomorphine-induced climbing behaviour is achieved by blocking either or both dopamine D_1_ and D_2_ receptors. Apomorphine induces a peculiar climbing behaviour in mice characterized initially by rearing and then spontaneous climbing activity. In the present study, acute pretreatment of AZE (100, 300, and 1000 mg/kg) in mice showed a significant but not dose-dependent decrease in frequency of climbing and climbing time induced by apomorphine compared to the vehicle-treated group. Haloperidol, the reference drug, significantly and dose-dependently reduced the climbing behaviour of mice as expected. AZE was less potent than HAL in reducing the apomorphine-induced climbing behaviour of mice as indicated by the ED_50_ values. However, the effects of AZE at dose of 300 mg/kg p.o. on climbing behaviour were comparable to haloperidol at dose of 1 mg/kg i.p. Based on these findings, it may be concluded that AZE produces antipsychotic activity which may be mediated through the antagonism of either or both of dopamine D_1_ and D_2_ receptors in the striatum of the brain.

Catalepsy test in rodents is a predictive tool for detecting the extrapyramidal side effects (EPS) of antipsychotic drugs [44]. EPS is a key side effect that is known to account for the discontinuation of antipsychotic drug use in patients [45]. Consequently, current research for new antipsychotic drug discovery is focused on agents with minimal or no EPS in addition to clinical efficacy [45, 46]. Catalepsy in laboratory animals is defined as a failure to correct an externally imposed posture and the test entails measuring the latency for the animal to remove itself from the unfamiliar and uncomfortable position [19, 47]. AZE's lack of catalepsy in the mice over the entire 60 min after administration period is an indication that it may not produce any significant EPS. However, this claim can be substantiated following the determination of the incidence of catalepsy after a chronic administration of the AZE. The extract, at the highest dose used in the study, produced a significant increase in haloperidol-induced catalepsy, suggesting it may have the ability to cause this side effect at higher doses.

The forced swimming and tail suspension tests are the two widely used animal models for antidepressant screening. They are quite sensitive and specific to all the major classes of antidepressant drugs [22]. They are sensitive to both acute and chronic treatments of antidepressants [48]. The TST has a higher pharmacological sensitivity than the FST and it is also less stressful [49]. In both tests, mice are placed in an inescapable but moderately stressful situation. Lack of escape related behaviour is considered immobility and it reflects a state of despair which is claimed to produce in the animals a condition similar to depression in humans. This state of despair can be reduced by several agents which are therapeutically effective in human depression [48, 50]. Reduction of the immobility time has been established to depend predominantly on the enhancement of central 5‐HT and catecholamine neurotransmission [51]. In the present study, AZE failed to reduce the immobility time in the FST and TST and it therefore follows that AZE possesses no potential antidepressant activity.

Alkaloids, flavonoids, tannins, saponins, and anthraquinone glycosides were found to be present in AZE. Similarly, alkaloids, tannins, saponins, flavonoids, and cardiac glycosides in the stem bark of* Albizia zygia* have been reported [9]. The observed neuropharmacological effects of the plant extract could be attributed to the presence of these phytochemical constituents. However, activity-guided characterization of these groups would be needed to comprehend their role in the observed antipsychotic effects.

## 5. Conclusion

The extract of* Albizia zygia* produced antipsychotic-like effects in mice. It, however, did not have any antidepressant effect. These findings support the use of the plant extract in the traditional medicinal management of psychosis in Ghana.

## Figures and Tables

**Table 1:** ED_50_ values (mg/kg) ± SEM of AZE and haloperidol (HAL) on the frequency and total duration of apomorphine-induced cage climbing in mice.

	AZE	HAL
Frequency	979.4 ± 1.35	0.1391 ± 1.07
Duration	240.8 ± 0.46	0.1047 ± 1.61

**Table 2:** Effect of extracts AZE, haloperidol (HAL), and vehicle on the duration of catalepsy. Data are mean ± SEM (*n* = 5). ^*∗*^*P* ≤ 0.05, ^*∗∗*^*P* ≤ 0.01, compared with vehicle group (two-way ANOVA followed by a Bonferroni's post hoc test).

Treatment (mg/kg)	Time (s)			*P* value
	15th min	30th min	60th min	
Vehicle	0.000 ± 0.00	0.00 ± 0.00	0.00 ± 0.00	—

AZE 100	0.000 ± 0.00	1.398 ± 0.87	1.398 ± 1.09	0.23
AZE 300	0.000 ± 0.00	0.200 ± 0.13	1.300 ± 1.07	
AZE 1000	0.400 ± 0.40	0.000 ± 0.00	0.000 ± 0.00	

HAL 0.3	34.187 ± 8.01	87.902 ± 33.81	108.892 ± 32.84	0.01
HAL 1.0	154.340 ± 42.16^*∗∗*^	153.273 ± 52.08^*∗*^	125.403 ± 55.14^*∗*^	
HAL 3.0	162.660 ± 46.41^*∗∗*^	179.510 ± 50.24^*∗∗*^	192.122 ± 40.87^*∗∗*^	
